# A Genetic Screen for Anchorage-Independent Proliferation in Mammalian Cells Identifies a Membrane-Bound Neuregulin

**DOI:** 10.1371/journal.pone.0011774

**Published:** 2010-07-26

**Authors:** Davide Danovi, Catherine A. Cremona, Gisela Machado-da-Silva, Sreya Basu, Luke A. Noon, Simona Parrinello, Alison C. Lloyd

**Affiliations:** MRC Laboratory for Molecular Cell Biology and The UCL Cancer Institute, University College London, London, United Kingdom; University of Birmingham, United Kingdom

## Abstract

Anchorage-independent proliferation is a hallmark of oncogenic transformation and is thought to be conducive to proliferation of cancer cells away from their site of origin. We have previously reported that primary Schwann cells expressing the SV40 Large T antigen (LT) are not fully transformed in that they maintain a strict requirement for attachment, requiring a further genetic change, such as oncogenic Ras, to gain anchorage-independence. Using the LT-expressing cells, we performed a genetic screen for anchorage-independent proliferation and identified Sensory and Motor Neuron Derived Factor (SMDF), a transmembrane class III isoform of Neuregulin 1. In contrast to oncogenic Ras, SMDF induced enhanced proliferation in normal primary Schwann cells but did not trigger cellular senescence. In cooperation with LT, SMDF drove anchorage-independent proliferation, loss of contact inhibition and tumourigenicity. This transforming ability was shared with membrane-bound class III but not secreted class I isoforms of Neuregulin, indicating a distinct mechanism of action. Importantly, we show that despite being membrane-bound signalling molecules, class III neuregulins transform via a cell intrinsic mechanism, as a result of constitutive, elevated levels of ErbB signalling at high cell density and in anchorage-free conditions. This novel transforming mechanism may provide new targets for cancer therapy.

## Introduction

Most normal mammalian cells require both mitogens and anchorage signals in order to proliferate, and are sensitive to anti-proliferative cues from surrounding cells, a process known as contact inhibition. In contrast, proliferation in the absence of mitogens, loss of contact inhibition and anchorage-independent proliferation are thought to be key characteristics of cancer cells [1] with anchorage-independent proliferation a powerful predictor of tumourigenic and metastatic potential [2]. Previous studies in several primary cell types have demonstrated that multiple genetic changes are required to enable anchorage-independent proliferation. These cooperative events commonly involve inactivation of the Rb and p53 tumour suppressor pathways together with activation of Ras signalling pathways [3].

Primary rat Schwann cells can be passaged indefinitely in culture while maintaining normal cell-cycle checkpoints [4]. Previous characterisation of the oncogenic pathways required to transform these cells showed that, as for other cell types, expression of SV40 Large T antigen (LT), which inactivates the p53 and Rb pathways, permits the cells to proliferate mitogen-independently but they retain a strict requirement for attachment and exhibit contact inhibition of proliferation. However, coexpression of oncogenic Ras allows both proliferation in the absence of anchorage and at high cell densities [5]. Consistent with these findings, Schwann cell tumours frequently have defects in the p53 and Rb pathways and activation of the Ras pathway, induced for example by loss of the NF1 gene [6], [7].

To identify genes capable of inducing anchorage-independent proliferation and thus potential new targets for cancer therapy, we established a cDNA retroviral screen in LT-expressing Schwann cells. From this screen, we isolated the SMDF isoform of Neuregulin 1 (NRG1). Neuregulins are a large family of EGF-like ligands involved in cell-cell communication in many different cell types [8], [9]. The complexity of NRG1 signalling is partly the result of a large number of alternatively-spliced forms that signal in distinct ways [10]. The class I and II forms of NRG1 act as classic soluble factors whereas class III isoforms, of which SMDF is a member, remain anchored to the membrane and signal to neighbouring cells in a juxtacrine manner. Class III members of the NRG1 family play a pivotal role in the life of a Schwann cell. Expressed by axons, they signal to Schwann cells by direct contact, promoting proliferation of the progenitor cells during development then differentiation and myelination at later stages [11], [12], [13], [14], [15]. Here we find that class III members of this family - including SMDF, but not class I secreted forms - when expressed directly by Schwann cells promote oncogenic transformation. This cell-intrinsic behaviour appears to be the result of constitutive neuregulin signalling at high density and in the absence of anchorage. The oncogenic activity of class III isoforms of neuregulin identifies a further role for this family of signalling molecules in the development of cancer.

## Results

### A screen for anchorage-independent proliferation

Schwann cells expressing LT (NSLT) are strictly dependent on attachment signals for proliferation, yet only require a single additional change, such as oncogenic Ras expression, to proliferate anchorage-independently [5]. Moreover, as these cells have an extremely low rate of spontaneous transformation they are an ideal cell system for a screen aimed at the isolation of novel genes promoting anchorage-independent proliferation. For the screen, we infected 20 million NSLT with either a retroviral cDNA expression library derived from human fetal brain or the same parental vector expressing GFP as a negative control. We chose this library since overexpression is a common method of oncogene activation in cancer cells and fetal brain is likely to contain cells with stem-like proliferative potential. After infection, cells were plated in soft agar suspension and screened for colony formation. As expected, no colonies formed in GFP cells. In contrast, from cells infected with the library, we retrieved 4 colonies. Importantly, cells dissociated and expanded from all isolated colonies maintained anchorage-independent proliferation when replated in soft agar (not shown).

**Figure 1:**
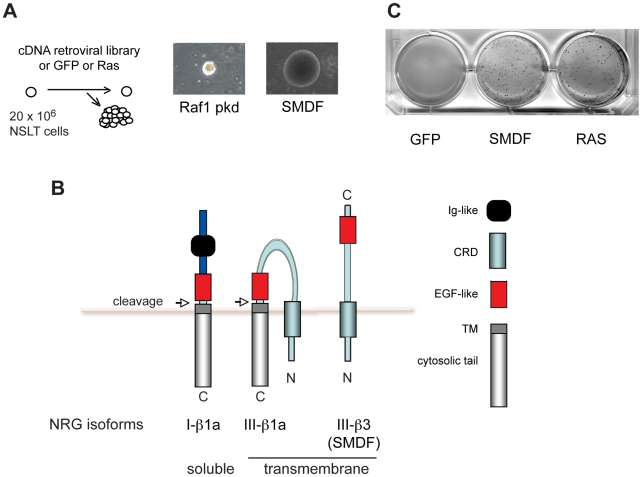
A screen for genes promoting anchorage-independent proliferation identifies SMDF. (A) Schematic of screening strategy and phase-contrast images of colonies formed after 3 weeks in soft agar from which the indicated inserts were isolated. Raf1 pkd  =  protein kinase domain of Raf1. (B) Schematic of the structure of the different forms of Neuregulin1 investigated in our study. CRD =  cysteine-rich domain. TM =  transmembrane region. (C) NSLT infected with Ras, SMDF or control vector were plated in suspension in soft agar and colonies stained after 3 weeks.

From the 4 colonies, 7 inserts were amplified by PCR. These were sequenced and re-cloned into the original retroviral vector. Of these, only 2 induced anchorage-independent proliferation when reintroduced into NSLT (Figure 1A). One of these inserts contained the protein kinase domain of c-Raf1. c-Raf1 is an immediate downstream target of the Ras pathway with known transforming activity [16] and therefore confirmed the validity of the screen and suggested that in Schwann cells, Ras acts through Raf to induce anchorage independent proliferation.

### NRG1 SMDF promotes oncogenic transformation

**Figure 2:**
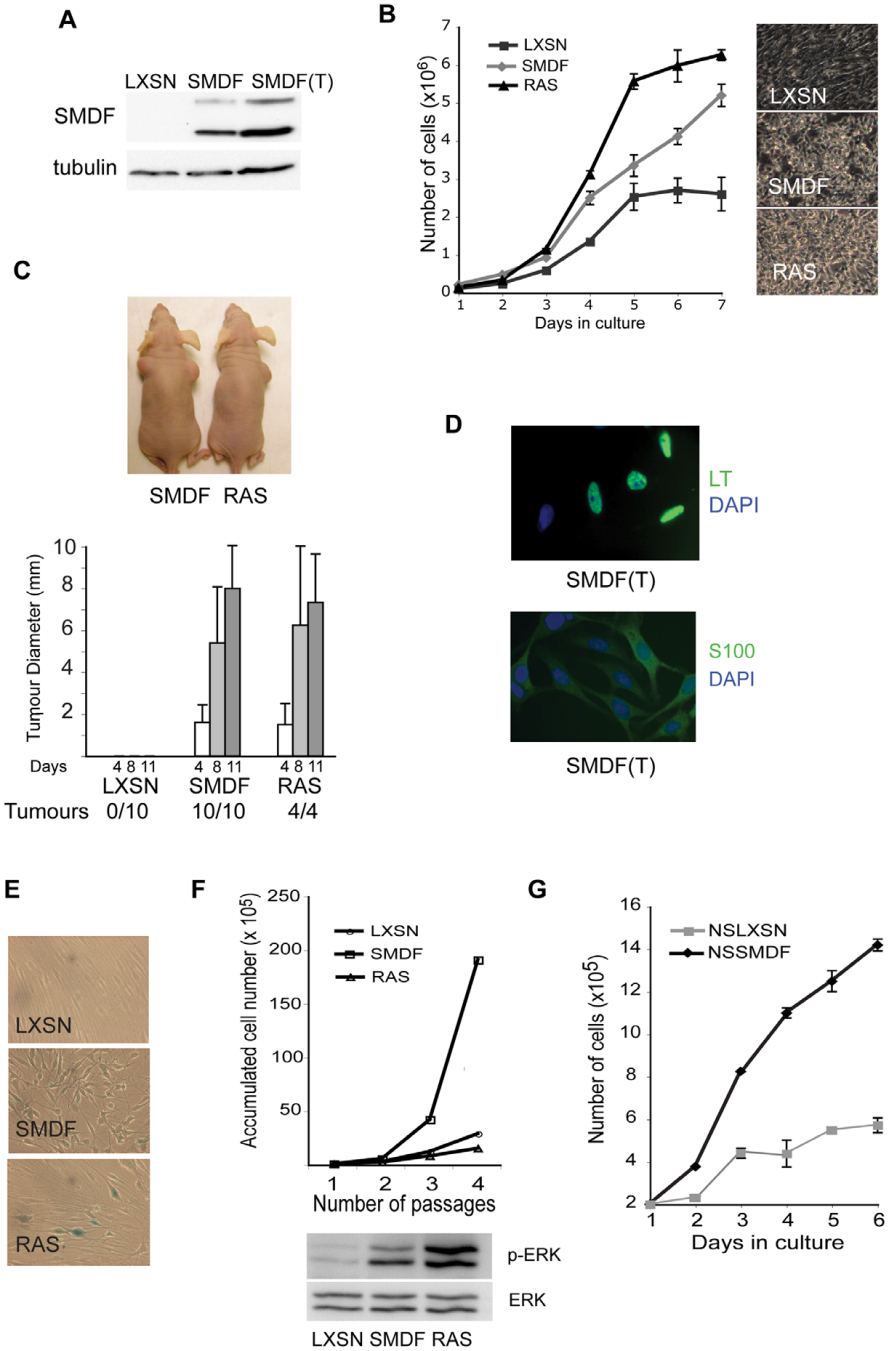
SMDF promotes oncogenic transformation. (A) Western blot analysis of total lysates from NSLT cells selected to express human SMDF or control vector (LXSN). SMDF(T) are derived from tumours, induced by injecting NSLTSMDF cells into nude mice. (B) Cell counts −/+ S.D. following seeding of 10^5^ NSLT cells per well expressing SMDF, Ras or control vector (LXSN) in triplicate in 6 well plates. Medium was changed daily. Experiment shown is representative of 3 separate experiments. Representative phase-contrast images of the cells at high density. (C) NSLT cells expressing SMDF, Ras or control vector (LXSN) were injected into the flanks of nude mice. Ras cells were only injected into a single flank. Picture shows examples of tumours formed (SMDF (left), Ras (right)) and the graph shows the average growth of the tumours −/+ S.D.. (D) Representative images of cells dissociated from NSLTSMDF derived tumours stained for LT (upper panel) or the Schwann cell marker, S100 (lower panel). Primary Schwann cells (NS) were infected with retroviral vectors expressing SMDF, Ras or the control vector (LXSN) and selected in G418. One week following selection, the cultures were fixed and stained for the senescence marker β-Galactosidase at pH 6 (E) or seeded in triplicate and counted for four passages (F). Western blot shows p-ERK levels in lysates prepared from NS cells expressing Ras, SMDF or control vector (LXSN) six days after infection. (G) 2×10^5^ NS cells infected with SMDF or control vector (LXSN) were plated in triplicate in 6 well dishes and counted at the indicated days. Medium was changed daily. Error bars show −/+ S.D..

The other insert isolated in the screen encoded the full length SMDF isoform of NRG1. SMDF (NRG1 III-β3) is a transmembrane member of the NRG family of signalling molecules [10], [17] and is similar to the cleaved form of the more commonly expressed NRG1 III-β1a. (Figure 1B). When plated in soft agar, SMDF but not GFP infected NSLT formed colonies in suspension with a similar efficiency to Ras infected NSLT indicating that SMDF is contributing to transformation directly and not through additional mutation (Figure 1C). Loss of contact inhibition is a distinct property of transformed cells, so we tested the ability of SMDF expression to stimulate proliferation at confluence. We retrovirally infected and selected pools of NSLT cells with SMDF, Ras or empty vector (Figure 2A), seeded the cells at sub-confluency and then counted cells at different times after plating (Figure 2B). NSLT infected with empty vector slowed their proliferation rate around confluence whereas SMDF-expressing cells, like those expressing Ras, kept proliferating reaching a higher cell density. To assess whether SMDF infected NSLT were tumourigenic we inoculated LXSN, SMDF or Ras infected NSLT into the flanks of nude mice. Strikingly, in less than 2 weeks, all SMDF (10/10) and Ras (4/4) inoculations resulted in tumours with comparable growth rates (Figure 2C). In contrast, no NSLT infected with empty vector LXSN (0/10) gave rise to tumours. To confirm that the tumours arose from LT Schwann cells expressing SMDF, we dissociated and cultured cells from 3 SMDF tumours. Immunofluorescence analysis showed that the majority of these cells retained expression of the Schwann cell marker S100 and LT antigen (Figure 2D) confirming that the tumours were derived from NSLT. Moreover, SMDF expression was maintained (Figure 2A). As expected, these cells also maintained the capacity to proliferate in suspension (not shown). Together these results show that SMDF cooperates with LT to induce full oncogenic transformation.

### SMDF expression confers a proliferative advantage to normal Schwann cells

Oncogenic Ras is transforming in the presence of additional genetic changes. When expressed alone in primary cells however, oncogenic Ras induces premature senescence, a putative tumour suppressor mechanism to protect from uncontrolled proliferation [18]. To address the effects of SMDF expression in normal cells, we infected primary Schwann cells with Ras (NSRas), SMDF (NSSMDF) or an empty LXSN vector control (NSLXSN) and isolated pools of neomycin resistant cells. Following drug selection, a clear phenotypic change was observed in both the NSRas and NSSMDF cells. NSRas cells developed a classical senescent phenotype with large multinucleated cells that stained positively for the senescence marker β-galactosidase at pH 6.0 (Figure 2E). Moreover these cells could not be expanded in culture unlike control cells (Figure 2F). In contrast, NSSMDF cells developed a refractile appearance, proliferated more rapidly than control cells and did not undergo premature senescence as judged by staining negatively for β-galactosidase and by readily expanding in culture (Figure 2E and 2F). It has previously been shown that the ERK signalling pathway mediates Ras-induced senescence [19], [20], [21]. Consistent with this, Ras expression induced higher levels of P-ERK compared to that seen in SMDF expressing cells (Figure 2F). NSSMDF cells however, had lost certain proliferative controls in that they continued to proliferate at confluency, reaching a higher density than control cells (Figure 2G). Together these results show that SMDF expression, unlike oncogenic Ras, fails to induce senescence but instead promotes deregulated proliferation of normal primary cells. These cells are not fully transformed however, as they are unable to form colonies in soft agar (not shown). Nevertheless, the fact that overexpression of SMDF does not induce premature senescence could make it a favourable early event in tumourigenesis.

### SMDF transforms via ErbB receptor signalling

**Figure 3:**
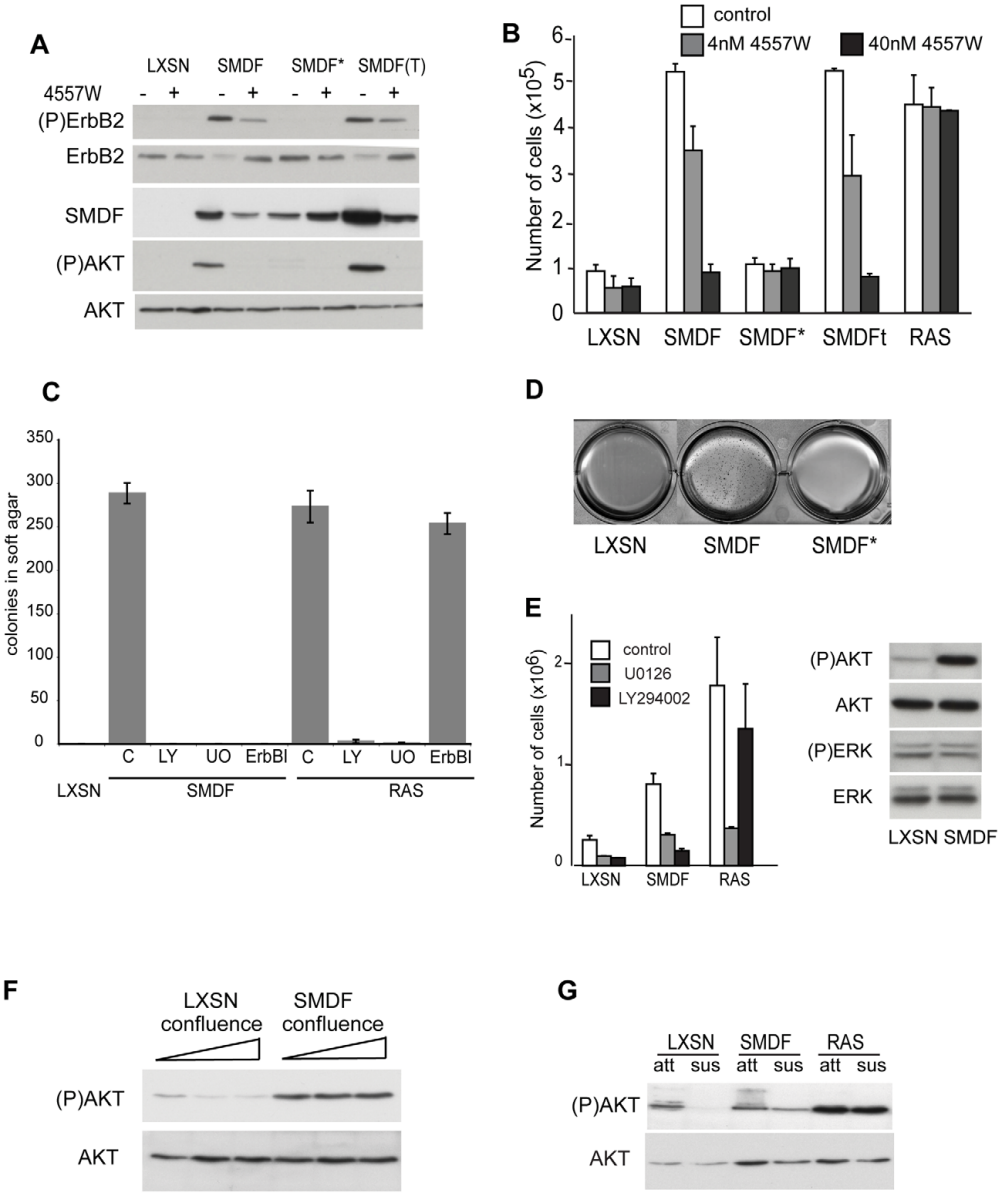
SMDF transforms via ErbB receptor signalling. (A) Western blot analysis of indicated proteins in NSLT cells expressing SMDF, SMDF* (point mutant incapable of binding ErbB receptors) or control vector (LXSN), and SMDF(T) cells derived from SMDF tumours in the absence or presence of the ErbB-2 inhibitor, 4557W (40 nM). (B) Cell counts of triplicate wells in the presence or absence of the indicated concentrations of 4557W −/+ S.D.. Figure is representative of 3 independent experiments. (C) Number of colonies formed in soft agar assays of NSLT cells expressing SMDF, Ras or control vector LXSN in the absence or presence of the indicated inhibitors. U0126 (UO), LY294002 (LY) and the ErbB inhibitor (ErbBI). 12 fields were counted per well with the results shown as average/well −/+ S.D.. Results are representative of two separate experiments (D) Soft agar assays of NSLT cells infected with SMDF, SMDF* or control vector (E) 10^5^ NSLT cells expressing SMDF, Ras or control vector (LXSN) were seeded in triplicate into 6 well dishes and counted at 72 h. The inhibitors U0126 (20 µM), LY294002 (20 µM) or control vehicle were added where indicated at 36 hours. Western blot analysis of total lysates from NSLT cells expressing SMDF or control vector (LXSN). (F) Western blot analysis of total lysates from NSLT cells expressing SMDF or control vector (LXSN) collected 3 (preconfluent), 6 and 9 days after seeding. Plates were medium changed daily. (G) Western blot analysis of total lysates from NSLT cells expressing SMDF, Ras or control vector (LXSN) in attached (att) or anchorage-independent conditions (sus).

NRG1 isoforms share an EGF domain through which they bind to and activate the ErbB family of receptors [10]. In Schwann cells, it has been shown that ErbB-2/ErbB-3 receptor heterodimers mediate neuregulin signalling as Schwann cells do not express ErbB-1 and ErbB-4 [14], [22]. Increased levels of ErbB-2 receptor phosphorylation were detectable in NSLTSMDF cells and the SMDF expressing cells isolated from tumours (SMDFT) when compared to control cells (Figure 3A) indicating that receptor signalling was activated in these cells. Total ErbB-2 levels appeared somewhat lower in SMDF expressing cells than in LXSN infected cells consistent with previously reported ligand-induced degradation of the receptor [23]. To address whether ErbB-2/ErbB-3 signalling was responsible for SMDF transforming activity we took two distinct approaches. First, we determined the effects of ErbB inhibitors on SMDF induced transformation. Using the cell-permeable, ErbB-specific inhibitor 4557W [24] we found that the proliferation at confluency of SMDF infected cells and cells dissociated from SMDF tumours was selectively blocked by the inhibitor in a dose-dependent manner (Figure 3B). As expected, the levels of phosphorylated ErbB-2 receptor were strongly downregulated in these cells when treated with inhibitor (Figure 3A). In contrast, no significant differences in the number and morphology of cells were observed in the presence of the inhibitor for control cells and importantly in Ras infected NSLT. To confirm these findings, we repeated the cell density experiments with two separate ErbB-2 inhibitors and got similar results (Figure S1). To address whether ErbB-2/-3 signalling was responsible for the ability of SMDF to induce anchorage-independent proliferation, we added ErbB inhibitor to the soft agar assays and found that while it had no effect on the ability of Ras expressing NSLT cells to form colonies it completely blocked NSLTSMDF colony formation (Figure 3C). As a second approach, we made a point mutation in the SMDF EGF domain, which has previously been shown to disrupt binding to ErbB receptors (here referred to as SMDF*) [25]. The expression of SMDF* in NSLT cells failed to induce proliferation at confluence (Figure 3B) and anchorage independent proliferation (Figure 3D), despite comparable levels of expression (Figure 3A). Together these results show that SMDF exerts its transforming activity through ErbB-2/ErbB-3 receptors.

ErbB-2/ErbB-3 signalling is known to stimulate proliferation through the ERK and PI3-K signalling pathways [22]. To determine the role of these signalling pathways we treated preconfluent NSLT and NSLTSMDF with the MEK inhibitor U0126 and the PI3-K inhibitor LY 294002. Both inhibitors blocked the ability of the SMDF expressing cells to proliferate at confluency (Figure 3E). Moreover, both inhibitors blocked the ability of NSLTSMDF cells to form colonies in soft agar (Figure 3C) indicating that both of these signalling pathways are important for the transforming activity of SMDF. In contrast, the proliferation of Ras-expressing cells was much less sensitive to PI3-K inhibition at confluency although they also required this pathway to proliferate in soft agar. However, when we analysed the levels of activation of these signalling pathways we detected a dramatic activation of signalling by the PI3-K pathway as determined by high levels of p-AKT in the SMDF-expressing cells but could not reproducibly detect any increase in the levels of phosphorylated ERK (Figure 3E), although we could detect elevated levels in the NSSMDF cells (Figure 2F). These results are consistent with the reported strong signalling to the PI3-K pathway by the ErbB-2/-3 heterodimer yet indicate that signalling through both pathways is required for transformation [22].

SMDF expressing NSLT are insensitive to contact inhibitory signals and proliferate in the absence of anchorage. In normal cells, both of these cell-cycle checkpoints are reported to involve mechanisms by which mitogens can no longer activate intracellular signalling pathways [26], [27]. Consistent with these findings, the addition of mitogens to NSLT cells, at increasing cell densities and in the absence of anchorage, was unable to stimulate PI3-K signalling. In contrast, NSLTSMDF cells maintained high PI3-K signalling both at high density and in the absence of anchorage (Figure 3F and G).

### NRG1 class III but not class I isoforms are transforming

**Figure 4:**
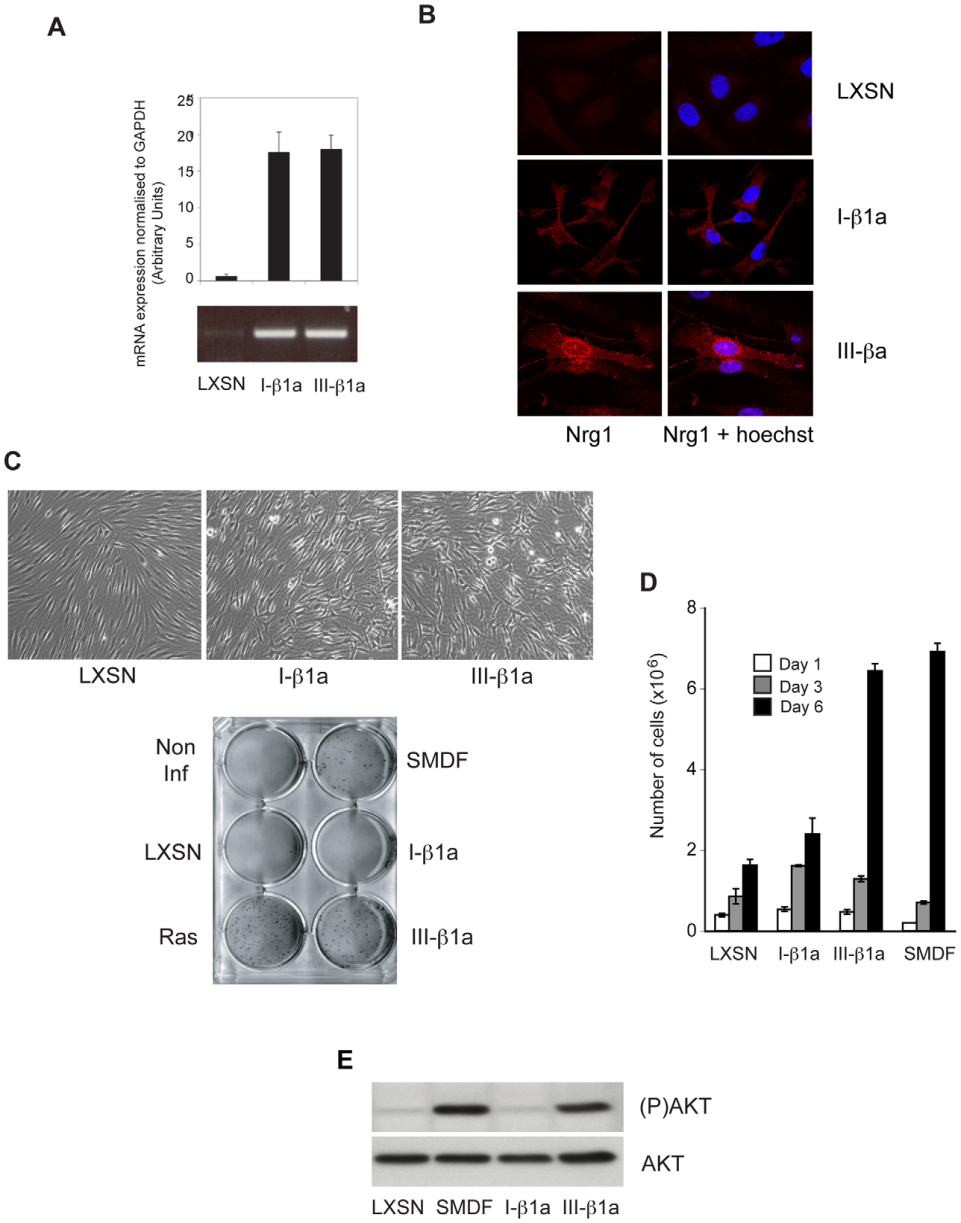
Transmembrane Class III Neuregulin 1 isoforms are transforming. (A) QPCR analysis of the mRNA levels and (B) immunostaining of NRG1 I-β1a and NRG1 III-β1a infected NSLT cells. (C) Phase contrast micrographs of NSLT infected with NRG1 I-β1a, NRG1 III-β1a or control vector (LXSN). The same cells together with SMDF and Ras- expressing cells were plated into soft agar and colonies stained after 3 weeks (C) or plated in triplicate and counted at indicated times (D) (Results shown are mean −/+ S.D.). (E) Western analysis of total lysates prepared from confluent LT cells expressing NRG1 I-β1a, NRG1 III-β1a, SMDF or control vector.

NRG1 class I isoforms act as classic soluble EGF-like factors whereas class III isoforms signal to neighbouring cells in a juxtacrine fashion. SMDF belongs to class III and shares with other members of this class a cysteine-rich domain (CRD) that anchors the protein to the cell surface [10]. To address whether the transforming activity was restricted to SMDF, we expressed two other well-characterised NRG1 isoforms in NSLT cells both of which are highly expressed in the PNS, from class I (I-β1a) or class III (III-β1a) [12] (Figure 1B). Detailed characterisation of these isoforms has shown that whilst the EGF-containing domain of NRGI I-β1a is efficiently shed from cells, including Schwann cells, the majority of NRG1 III-β1a remains at the cell surface [28]. RT-PCR analysis showed that the two isoforms were expressed at comparable levels in the NSLT cells (Figure 4A) and immunocytochemistry detected similar levels of protein within the cells (Figure 4B). The NRG1 I-β1a and NRG1 III-β1a expressing cells exhibited a similar transformed phenotype with cells more spindly and refractile than vector control cells (Figure 4C). However, only the class III isoform efficiently induced colony formation in NSLT and was capable of overcoming contact inhibition to allow cell proliferation at high densities (Figure 4C and 4D). Consistent with these results, only the class III isoforms stimulated high levels of PI3-K signalling at high cell densities (Figure 4E). Together these results indicate a specific role for class III as opposed to class I NRG1 isoforms in promoting oncogenic transformation.

### SMDF transforms NSLT cells via a cell-intrinsic mechanism

**Figure 5:**
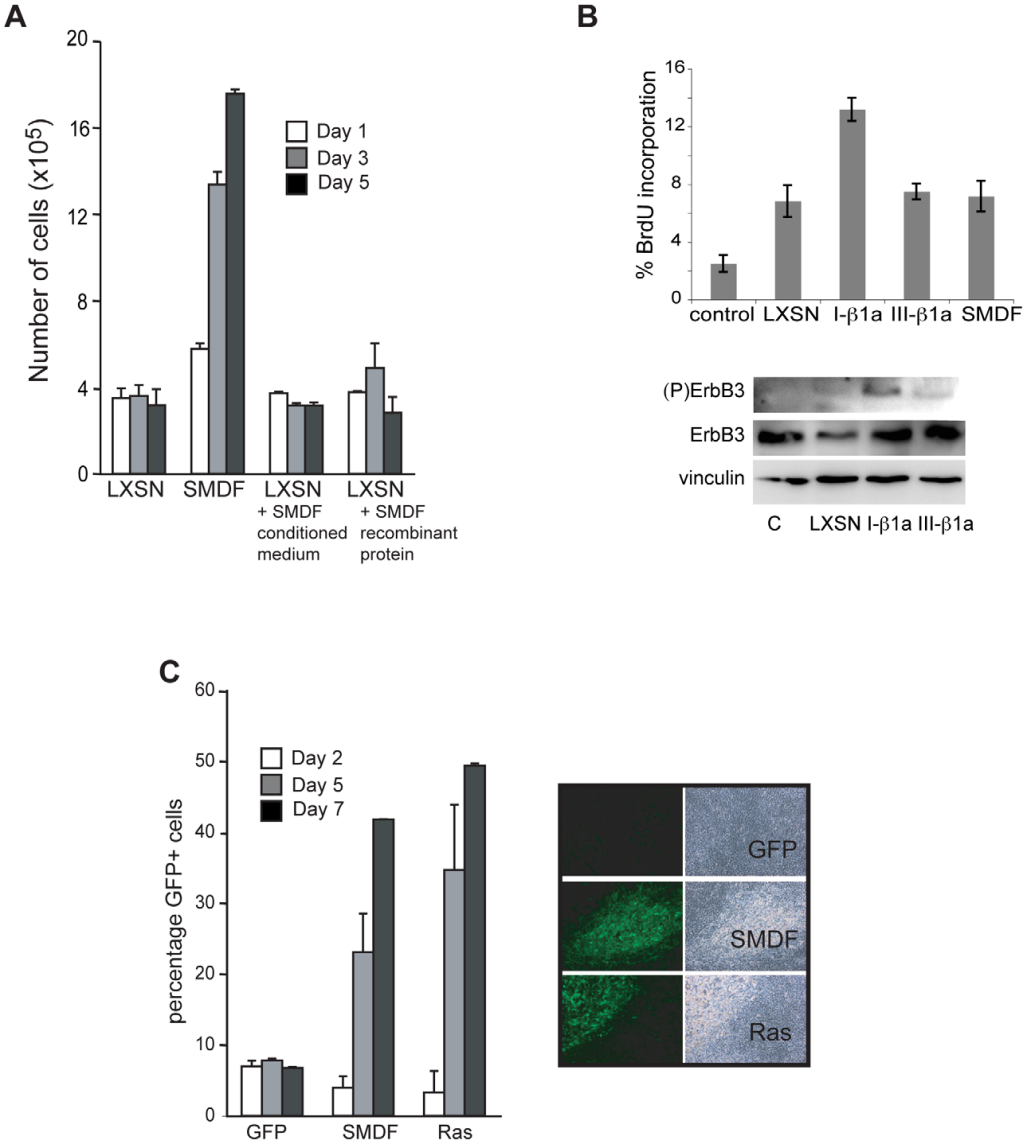
SMDF transforms by a cell-intrinsic mechanism. (A) Cell counts from triplicate wells of NSLT cells treated daily with conditioned medium from SMDF infected cells or recombinant SMDF (200 ng/ml). SMDF-infected NSLT cells were used as a positive control. Results shown are mean −/+ S.D. (B) Conditioned medium collected from confluent Schwann cells as indicated was used to stimulate Schwann cells in defined medium. Upper graphs shows BrdU incorporation of the stimulated cells (−/+ S.D.). Lower panel shows activation of the ErbB-2/-3 receptor as measured by levels of (P) ErbB-3. (C) NSLT cells were infected with SMDF, Ras or control vector GFP. The GFP-expressing cells were then mixed at a 1∶10 ratio with uninfected cells and plated in triplicate at sub-confluent density. At the times shown, the cells were trypsinised and reseeded and the percentage GFP-expression counted. Representative fluorescent and phase-contrast images are shown in the right hand panel (day 7).

A distinction between the class I and class III isoforms is that the class I molecules act as soluble factors whereas the class III molecules tend to signal by direct cell-cell contact. However, it has been reported that the transmembrane class III NRG1 isoforms can also be processed and released as soluble factors, albeit at low efficiency [8], [29]. To determine whether class III NRG1 isoforms exert their transforming activity as secreted, soluble factors, we asked whether the addition of conditioned medium from NSLTSMDF or high levels of recombinant soluble SMDF could transform NSLT cells. As shown in Figure 5A, the daily addition of either conditioned medium or high levels of soluble SMDF was unable to stimulate proliferation at high density. Similar results were obtained by adding the recombinant extracellular domain of NRG1 I-β1a or the recombinant EGF domain of NRG1 I-β1a (not shown). Moreover, the addition of recombinant factors to NSLTLXSN cells in soft agar was unable to promote colony formation (not shown). This was despite the ability of both recombinant SMDF and recombinant NRG1 I-β1a to stimulate DNA synthesis in quiescent Schwann cells (Figure S1C). Consistent with these results and previous studies [28], we were only able to detect mitogenic activity in conditioned medium from NRG1 I-β1a- expressing cells, which correlated with activation of the ErbB-2/B-3 receptor complex in the stimulated Schwann cells (Figure 5B). Together these results suggest that SMDF is not acting as a soluble factor to mediate its transforming effect. SMDF could therefore signal in a juxtacrine manner to neighbouring cells, or drive a cell-intrinsic mechanism inducing proliferation only in SMDF-expressing cells. To distinguish between these possibilities, we used a vector driving the expression of GFP together with SMDF. We set up a series of experiments using mixed populations of cells, GFP-positive expressing an empty vector, SMDF or Ras, and GFP-negative controls. We then assessed the percentage of GFP-positive cells at different times after plating at high density - conditions in which control cells fail to proliferate. If the GFP-positive cells overexpressing SMDF proliferate faster than the GFP-negative control cells, we would expect the percentage of GFP-positive cells to increase over time. Figure 5C shows that when approximately 1 in 10 of the cells were initially GFP-positive and coexpressing SMDF or Ras, the percentage of GFP-positive cells increased with time until, by 7 days, the GFP cells constituted greater than 40% of the cell population. Similar results were obtained starting from a 1∶1 ratio of GFP positive and GFP-negative cells (not shown). The proliferation rate of GFP negative cells was comparable in all samples tested (Figure 5C and not shown). These results indicate that SMDF transforms cells by a cell intrinsic mechanism.

## Discussion

We report here a retroviral cDNA screen for genes promoting anchorage-independent proliferation in a mammalian cell model system. Rat Schwann cells expressing SV40LT proliferate in the absence of mitogens but retain other cell-cycle checkpoints such as anchorage dependence and contact inhibition of proliferation. However, they require only one additional genetic lesion (for example, oncogenic Ras) for complete neoplastic transformation [5]. Despite the loss of p53 and Rb checkpoints, Schwann cells expressing LT are genetically stable, with an extremely low rate of spontaneous transformation [30]. These cells are thus a useful mammalian model system to identify potential oncogenes. The validity of this approach was confirmed by the isolation of an insert expressing the protein kinase domain of a known oncogene, *RAF1*, which is known to have transforming activity [16] and the isolation of a candidate oncogene, SMDF, a transmembrane isoform of NRG1. It is likely that this screening protocol would be useful for identifying further oncogenes using alternative libraries and/or larger screens.

In Schwann cells expressing LT, we found that SMDF was sufficient to confer full cellular transformation: similarly to oncogenic Ras expression, the expressing cells grew to higher densities, proliferated in the absence of anchorage and formed tumours in immuno-compromised mice. However, when expressed in primary Schwann cells, SMDF and oncogenic Ras expression had distinct effects, in that oncogenic Ras induced cellular senescence, a checkpoint response thought to protect against the development of cancer, whereas SMDF expression conferred a proliferative advantage. It is known that cellular senescence induced by oncogenic Ras is mainly mediated by excessive signalling through the ERK signalling pathway[19], [20], [21]. Consistent with this, lower levels of ERK signalling was detected in SMDF-expressing cells compared to Ras-expressing cells. Also indicative of differential signalling, we found that confluent SMDF expressing LT cells were sensitive to inhibition of the PI3-K pathway whereas Ras-expressing cells were relatively unaffected. However, both the PI3-K pathways and the ERK pathways were required for SMDF-induced transformation. The observation that SMDF expression gave a proliferative advantage to primary Schwann cells without triggering the cellular “damage” response might indicate that SMDF expression could be a favourable early event in Schwann cell tumourigenesis. Interestingly, whilst oncogenic Ras is rarely found in Schwann cell derived tumours, SMDF and related family members appear to be expressed in a number of these tumour types [31], [32], [33].

SMDF is a member of a large family of secreted and membrane-bound factors produced by alternative splicing of the NRG1 gene. Despite their differences, what is common to the isoforms is that they signal, via an EGF domain, to activate the ErbB family of receptors. Consistent with this known mechanism of action, we found that SMDF-transforming activity requires signalling through the ErbB-2 receptor. However, despite the ability of all NRG isoforms to activate this receptor, we found that not all isoforms were equally transforming. In particular, a secreted class I isoform was inactive in our assays, even though these cells exhibited a transformed morphological phenotype. A property unique to the class III isoforms is that they normally signal as membrane-bound ligands suggesting that this may be important for their transforming activity. Consistent with this idea, adding high levels of a recombinant soluble form of SMDF was unable to confer any transforming ability. Interestingly, in line with our findings, it has been shown that transmembrane (class III) isoforms of NRG1 are more commonly over-represented than soluble (class I) forms in Schwann cell tumours, suggesting that differences between the isoforms may be relevant for tumour formation in vivo [32], [33], [34].

Class III isoforms of NRG1 normally signal in a juxtacrine fashion to neighbouring cells however, we have found that SMDF transforms by an intrinsic mechanism. It is not clear whether membrane-bound SMDF activates ErbB-2/ErbB-3 at the plasma membrane or whether signalling takes place on intracellular vesicles, as we have not been able to detect the activated receptor by immunostaining (unpublished observations). However, what is clear is that when SMDF is expressed in the same cell as its receptor, it leads to constitutive activation of the ErbB-2 receptor and sustained activation of downstream signalling pathways. Moreover, the signalling is maintained at high cell density and in the absence of anchorage, when normal mitogenic signalling is suppressed. This suggests the autocrine loop established by co-expression of the membrane-bound ligand and its receptor results in sustained signalling, which is not subject to the regulatory mechanisms that suppress signalling when cells get too dense or lose anchorage. Although we have found that class III isoforms of NRG1 are potent in their transforming activities, our work does not rule out the possibility that other isoforms of NRG1 may also contribute to tumourigenesis. Indeed class II isoforms of NRG1 have also found to be expressed in Schwann cell derived tumours [32], [34] and in a transgenic mouse model, expression of a class II isoform in Schwann cells led to mice developing hyperplasia with 60% progressing to develop tumours that resembled human malignant peripheral nerve sheath tumours [35]. Moreover, class I isoforms may also be transforming if expressed at higher levels. Our results do however, indicate a potency of the class III isoforms and a distinct mechanism of action, which prompts further study.

We have found that class III isoforms transform by the establishment of a cell-intrinsic autocrine loop. Intriguingly, a recent study demonstrates that SMDF can also contribute to tumourigenesis by acting in a juxtacrine fashion. In this study, the authors constructed a transgenic mouse, which specifically expressed SMDF in postnatal neurons [36]. This resulted in Schwann cell hyperplasia and predisposed mice to Schwann cell-derived tumours. Thus, NRG1 class III isoforms appear to be able to promote both cell autonomous and non- cell autonomous oncogenic transformation. It will be of great interest to determine both the role of these molecules in human tumourigenesis and their mechanism of action.

## Materials and Methods

### Retroviral constructs

A retroviral cDNA library from human fetal brain (Stratagene Viraport 973221) was expanded and prepared for transfection according to the manufacturer's instructions. The library vector backbone pFB containing a GFP insert (pFB-GFP, Stratagene) was used as a negative control for the screen. The SMDF cDNA identified in the screen was further subcloned into retroviral vectors LXSN (Clontech) and GFP containing Pinco (kind gift from Piergiuseppe Pelicci's laboratory). The NRG1 I-β1a and III-β1a isoforms were a kind gift from Doug Falls and Carla Taveggia and were subcloned into LXSN [28].

### Cell culture and retroviral transduction

Schwann cells isolated from the sciatic nerves of 7-day-old rats were purified and cultured as previously described [4]. All retroviral infections were performed using viral supernatants prepared from Phoenix 393T producer cells transiently transfected with the library/vector of interest for 48 h using Lipofectamine plus (Invitrogen). The cells to be infected were plated the previous day (2.7×10^4^ cells/cm^2^) and retroviral transduction was performed by incubation for 3 hours at 37°C/5% CO_2_ in the presence of filtered viral supernatant containing 8 µg/ml polybrene. Following infection the medium was changed and cells were left to proliferate for 48 h before drug selection or seeding into soft agar for the screen. Schwann cells stably expressing SV40 Large T antigen (NSLT) were obtained by pooling and expanding puromycin-resistant cells (0.5 µg/ml) one week after infection with pBabe-puro-SV40 (kind gift of James DeCaprio). Primary Schwann cells (NS) were infected with LXSN, LXSN-SMDF or LXSN-Ras and selected in G418 (0.6 mg/ml) for 1 week. Recombinant soluble human SMDF (378-SM), and the extracellular (377-HB) and EGF domains (396-HB) of human NRG1 I-β1a were purchased from R&D Systems and used at the indicated concentrations. To collect conditioned medium, indicated cells were cultured to confluency, washed twice in DMEM and cultured for a further 24 hours in defined medium (DMEM containing 100 µg/ml transferrin, 100 µg/ml BSA, 60 ng/ml progesterone, 50 ng/ml thyroxine, 50 ng/ml tri-iodothyrine, 16 µg/ml putrescine and 40 ng/ml selenium). The medium was then used to stimulate sub-confluent Schwann cells in defined medium. BrdU incorporation was performed as previously described [30].

### Screen for anchorage-independent proliferation

Low passage NSLT cells were infected with the retroviral cDNA library 48 h before seeding into soft agar (5×10^5^ cells per 15 cm dish), which consisted of 0.45% Seaplaque agarose in normal culture medium. Soft agar cultures were incubated at 37°C/5% CO_2_ for 3 weeks and fed weekly with fresh medium. Colonies were isolated using a pipette tip and briefly expanded in adherent cultures before isolation of genomic DNA using the DNAeasy kit (Qiagen). Primers pFB 5′ 5′-GGCTGCCGACCCCGGGGGTGG-3′ and pFB 3′ 5′-CGAACCCCAGAGTCCCGCTCA-3′ were used to amplify integrated viral DNA and the resulting amplicons were cloned directly into the Zero Blunt vector (Invitrogen) before sequencing with pFB and T7 primers. All inserts isolated were further subcloned into the pFB vector for further validation of their capacity to transform.

### Transformation Assays

To measure anchorage independent proliferation, cells were seeded in soft agar (as described above) at 3×10^3^ cells/well in 6 well dishes in triplicate. Resulting colonies were visualised by staining with MTT (Calbiochem). For the inhibitor studies, the inhibitors U0126 (20 µM), LY294002 (20 µM) and ErbBI (N-(4-((3-Chloro-4-fluorophenyl)amino)pyrido[3,4-d]pyrimidin-6-yl)2-butynamide (Calbiochem)) (20 µM) were added in 1 ml of medium at 5X the final concentration to the 4 ml agar wells. To assess the ability of cells to proliferate at confluence, the indicated number of cells/well were seeded in triplicate in 6 well dishes. Then at indicated time points, cells were trypsinised and counted using a Beckman Coulter counter. In mixed population experiments GFP positive cells were counted using a fluorescent microscope and the mean of 10 fields used to calculate the percentage. In vivo tumourigenesis assays were performed in CD1 nude mice (Charles River) as previously described [37]. Briefly, 2.5×10^6^ cells were injected subcutaneously into each flank of the animal. The animals were then monitored and sacrificed either when tumours reached 10 mm diameter or after a maximum of 35 days. Representative tumours were collected and cells dissociated with trypsin and expanded in culture. The senescence marker, SA-βGal was detected in NS cells as previously described [4], 1 week following retroviral infection.

Ethics statement: All animal work has been conducted according to relevant national (Home Office Guidelines) and international guidelines and was approved by the University College London ethics committee.

### RT-PCR

Total RNA was extracted with the RNeasyPlusMinikit (Qiagen) and 1 µg of RNA reverse transcribed using SuperScriptII Reverse Transcriptase (Invitrogen). Pan-Neuregulin primers 5′-GGCCTACTGCAAAACCAAGA-3′ and 5′-TGATGGGCTGTGGAAGTGTA-3′ were used to detect expression of Nrg1 isoforms and using the DyNAmo SYBRGreen qPCRKit (NewEngland Biolabs) and Opticon2 DNAengine (MJResearch) and relative expression values were obtained by normalizing to GAPDH 5′-TGCACCACCAACTGCTTAG-3′ and 5′-GGATGCAGGGATGATGTTC-3′.

### Antibodies, Western Blotting and Immunofluorescence

The following antibodies were used for western blotting: β-tubulin (Sigma T-4026), phosphotyrosine Clone PT-66 (Sigma), Total ErbB-2 C18 (Santa Cruz), (P)AKT (ser 473) (Cell Signalling), AKT (H-136 Santa Cruz), (P)ERK (Sigma M8159), ERK (Sigma M5670), human SMDF AF378 (R&D) ErbB3 and (P)ErbB-3 (Cell Signalling). To analyse protein expression levels in cells in suspension, cells were seeded into methylcellulose for 24 hours, then retrieved by centrifugation at 4°C and lysed as previously described [30]. Cells dissociated from tumours were seeded on coverslips and stained for LT (PAB419) or S100β (DAKO). NRG1 isoform expression was detected using an antibody to the common intracellular domain (SC348 Santa Cruz).

## Supporting Information

Figure S1 Cell counts of triplicate wells seeded at high density with NSLT cells expressing SMDF, Ras or control vector (LXSN) in the presence or absence of the ErbB-2 inhibitors (A) ErbBI (N-(4-((3-Chloro-4-fluorophenyl)amino)pyrido[3,4-d]pyrimidin-6-yl)2-butynamide at 20 µM (B) ErbB2 inhibitor (4-(3-Phenoxyphenyl)-5-cyano-2H-1,2,3-triazole) at 30 µM −/+ S.D.. DMSO was added as control solvent. (C) Recombinant SMDF (200 ng/ml) or recombinant Nrg1 I-β1a (40 ng/ml) was added to quiescent Schwann cells in defined medium in the presence of IGF1 (20 ng/ml). BrdU was added for 8 hours, 18 hours after stimulation. Results shown are mean of triplicate wells (−/+ S.D.).
(0.25 MB TIF)
