# Supplementary figures and images for: A Genetic Screen for Anchorage-Independent Proliferation in Mammalian Cells Identifies a Membrane-Bound Neuregulin
Source: PLoS One. 2010 Jul 26;5(7):e11774. doi: 10.1371/journal.pone.0011774 (PMC2909903; doi:10.1371/journal.pone.0011774)

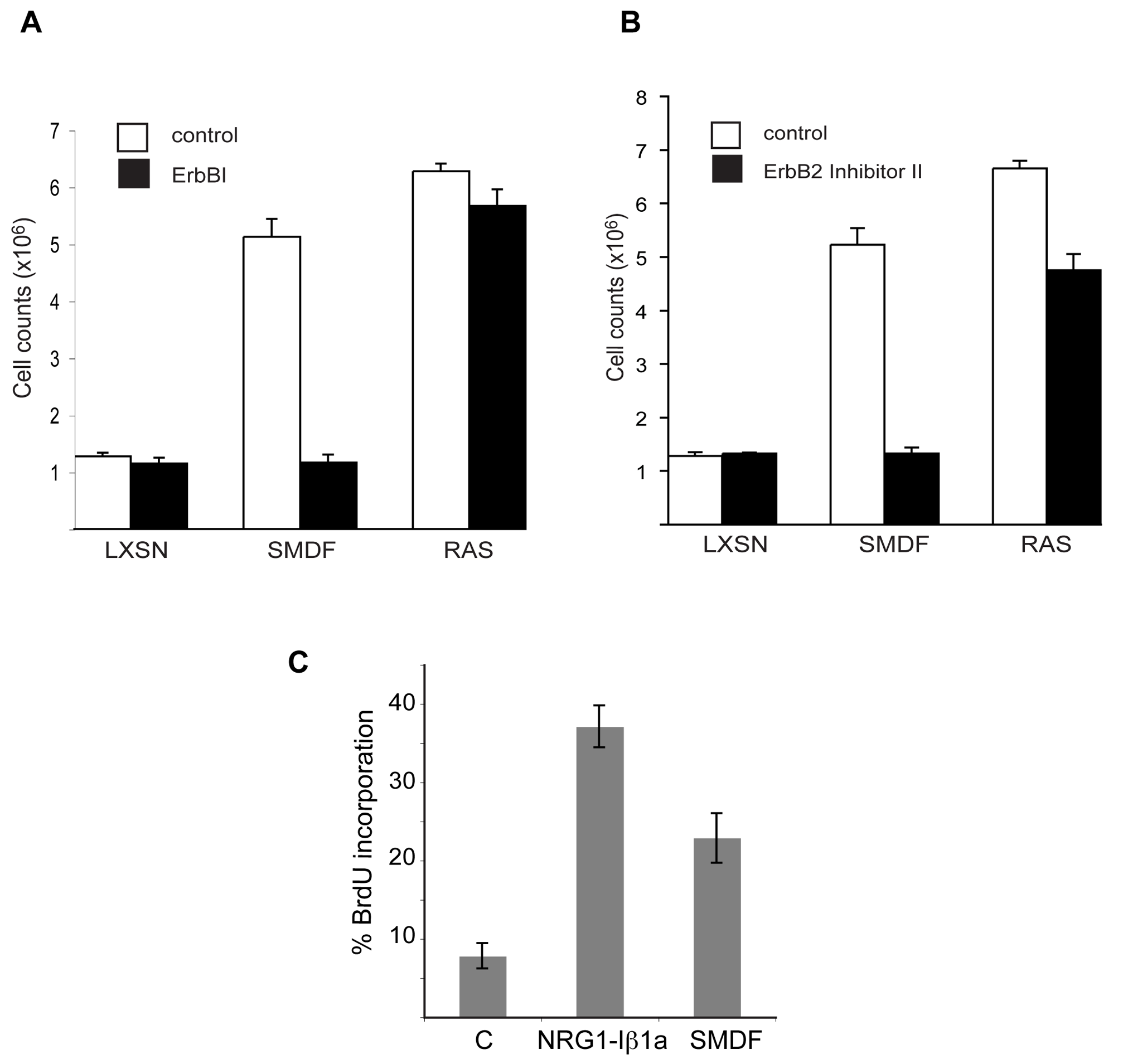

Supplement: Figure S1 — Cell counts of triplicate wells seeded at high density with NSLT cells expressing SMDF, Ras or control vector (LXSN) in the presence or absence of the ErbB-2 inhibitors (A) ErbBI (N-(4-((3-Chloro-4-fluorophenyl)amino)pyrido[3,4-d]pyrimidin-6-yl)2-butynamide at 20 µM (B) ErbB2 inhibitor (4-(3-Phenoxyphenyl)-5-cyano-2H-1,2,3-triazole) at 30 µM −/+ S.D.. DMSO was added as control solvent. (C) Recombinant SMDF (200 ng/ml) or recombinant Nrg1 I-β1a (40 ng/ml) was added to quiescent Schwann cells in defined medium in the presence of IGF1 (20 ng/ml). BrdU was added for 8 hours, 18 hours after stimulation. Results shown are mean of triplicate wells (−/+ S.D.). (0.25 MB TIF) [file pone.0011774.s001.tif]
